# Development and Formative Evaluation of a Virtual Exercise Platform for a Community Fitness Center Serving Individuals With Physical Disabilities: Mixed Methods Study

**DOI:** 10.2196/49685

**Published:** 2023-12-15

**Authors:** Sangeetha Mohanraj, Laurie A Malone, Christen J Mendonca, Mohanraj Thirumalai

**Affiliations:** 1 School of Health Professions The University of Alabama at Birmingham Birmingham, AL United States; 2 Department of Occupational Therapy School of Health Professions The University of Alabama at Birmingham Birmingham, AL United States; 3 Department of Health Services Administration School of Health Professions The University of Alabama at Birmingham Birmingham, AL United States

**Keywords:** web-based exercise, user experience, community health, fitness facility, tele-exercise, physical disability, physical activity, exercise, fitness, virtual, interface, disability, disabilities, accessibility, telehealth, telemedicine, eHealth, digital health, mixed methods study

## Abstract

**Background:**

People with disabilities experience numerous barriers to being physically active, such as transportation issues, a lack of trained exercise professionals who understand disabilities, and facility access. The use of a virtual exercise platform (VEP) may provide an alternative and limit certain barriers.

**Objective:**

The aim of this mixed method study was to evaluate user interaction (effectiveness, efficiency, and satisfaction), the strengths and weaknesses of the user interface, and the user experience with a VEP.

**Methods:**

Participants were recruited from a community fitness facility that offers programs for people with disabilities. Inclusion criteria were being older than 18 years, fluent in English, and availability of internet access. Features of the VEP included articles, prerecorded videos, live Zoom classes, web-based class registration, weekly progress tracking, incentives, and surveys. A one-on-one Zoom session was scheduled with each participant, during which they completed certain tasks: (1) create an account or login, (2) register for class, (3) join class, (4) add to calendar, and (5) complete surveys. As participants completed tasks, quantitative observations (time on task, task success, rate of task completion, and number of errors by users, which determined task difficulty), qualitative observations were made and interviews were conducted at the end of the session. The “concurrent think-aloud” method was encouraged by the moderator to gauge participants’ thoughts as they worked through testing. Participants also completed the System Usability Scale (SUS) and Questionnaire for User Interface Satisfaction (QUIS).

**Results:**

A total of 5 people with disabilities (3 male, 2 female), aged 36-78 (mean 54) years, with education levels from high school to PhD, were recruited. Devices used for testing included a laptop (n=3), a Chromebook (n=1), and a desktop (n=1). All participants completed tasks #1 and #2 without errors but could not complete task #4. One participant completed task #5 with difficulty and another completed task #3 with difficulty. The average time to complete each task was: (1) 82 seconds (55-110), (2) 11 seconds (4-21), (3) 9 seconds (5-27), and (4) 921.5 seconds (840-958). The mean SUS score was 86.5/100, and the mean user QUIS score was 8.08 out of 10. Qualitative observations indicated that the system was simple, user-friendly, and accessible.

**Conclusions:**

People with disabilities reported high usability and user satisfaction with the web-based exercise platform, and the system appears to be an efficient and effective physical activity option.

## Introduction

Exercise and physical activity have numerous beneficial effects on the physical and mental health and well-being of people [1]. These benefits can include a reduced risk of obesity, cardiovascular diseases, depression, and other severe health problems [2,3]. Physical activity for people with disabilities is even more important compared to people without disabilities, and people with disabilities have higher rates of obesity [4]. Additionally, people with disabilities have more barriers to being physically active due to a lack of trained exercise professionals who understand disability, accessible rehabilitation facilities, transportation, and so on [5,6].

Like many other industries, the COVID-19 pandemic impacted the fitness industry as well [7]. Due to COVID-19 safety concerns, various precautions such as social distancing, avoidance of other people, and stay-at-home orders to reduce exposure and the risk of illness became a necessity. Furthermore, individuals with underlying medical conditions and the elderly were more susceptible to developing COVID-19 complications [8,9]. As social distancing became a necessity, telehealth and other remote services became more prevalent, serving as a safer option to receive health care services in the home environment [10]. In April 2020, about 68% of members stated they were less likely to go back to the gym [11]. As of May 2020, more than 38,000 gyms were closed, and many gyms filed for bankruptcy, leading to job losses among physical trainers.

To continue serving their members, a community fitness facility in north central Alabama that provides opportunities for people with disabilities to achieve a healthy lifestyle through various recreation, physical activity, and health promotion activities devised new ways to deliver their programs. During the early stages of “lockdown,” program instructors began filming themselves teaching various fitness classes (eg, yoga and chair exercise) at home and posting them to the organization’s website. As the pandemic continued and eventually transitioned to restricted access, the organization began hosting several “live” Zoom classes that members could attend using a link provided via email.

As a next step to overcoming the pandemic impact, the fitness facility, in collaboration with the Rehabilitation Engineering Research Center on Recreational Technologies developed a virtual exercise platform (VEP). The platform provided the fitness facility with an opportunity to deliver their fitness programs in a more streamlined fashion and allowed members to continue being active throughout the pandemic.

## Mixed Methods Research in Usability

A mixed methods approach is advantageous to establishing the usability of a health application because researchers can consider multiple sources of data to identify and address specific areas for improvement. For this study, we implemented an iterative convergent mixed methods design proposed by Alwashmi et al [12]. Multiple cycles of simultaneous quantitative and qualitative data were collected and analyzed to address usability issues within this system. This design improves the usability of a health application by emphasizing integration across research aims, data collection, analysis, and interpretation [12].

The purpose of this study was to apply evidence-based user testing methods to evaluate user interaction with the VEP on International Organization for Standardization (ISO) criteria of effectiveness, efficiency, and satisfaction and identify strengths and weaknesses in the platform’s interface and user experience [13]. The mixed methods aim of this study was to explore the usability of the VEP and evaluate areas for improvement for the user. The quantitative aim of this study was to measure the platform’s effectiveness and efficiency, along with the user’s satisfaction. The qualitative aim was to characterize the user’s experience using the VEP and develop an understanding of their assessment. The goal was to generate ideas for improvements in the user’s experience to allow the platform to better accomplish its mission of helping people with disabilities participate in exercise programs and lead healthier lives. Due to time and resource limitations, the study scope was limited to three core functionalities of the platform: (1) setting up an account and registering for a class; (2) registering and completing preintervention surveys; and (3) registering for a class, adding it to the calendar, and joining the class. The objectives were to determine the following: (1) how accurately, quickly, and easily (effectiveness, efficiency, satisfaction) can a user set up an account and login? (2) How accurately, quickly, and easily (effectiveness, efficiency, and satisfaction) can a user register for the research study embedded in the system and answer the series of preintervention surveys? This is a secondary outcome to support future research. (3) How accurately, quickly, and easily (effectiveness, efficiency, and satisfaction) can a user register for a web-based class, add it to the calendar, and join the web-based class?

## Virtual Exercise Platform

**Figure 1:**
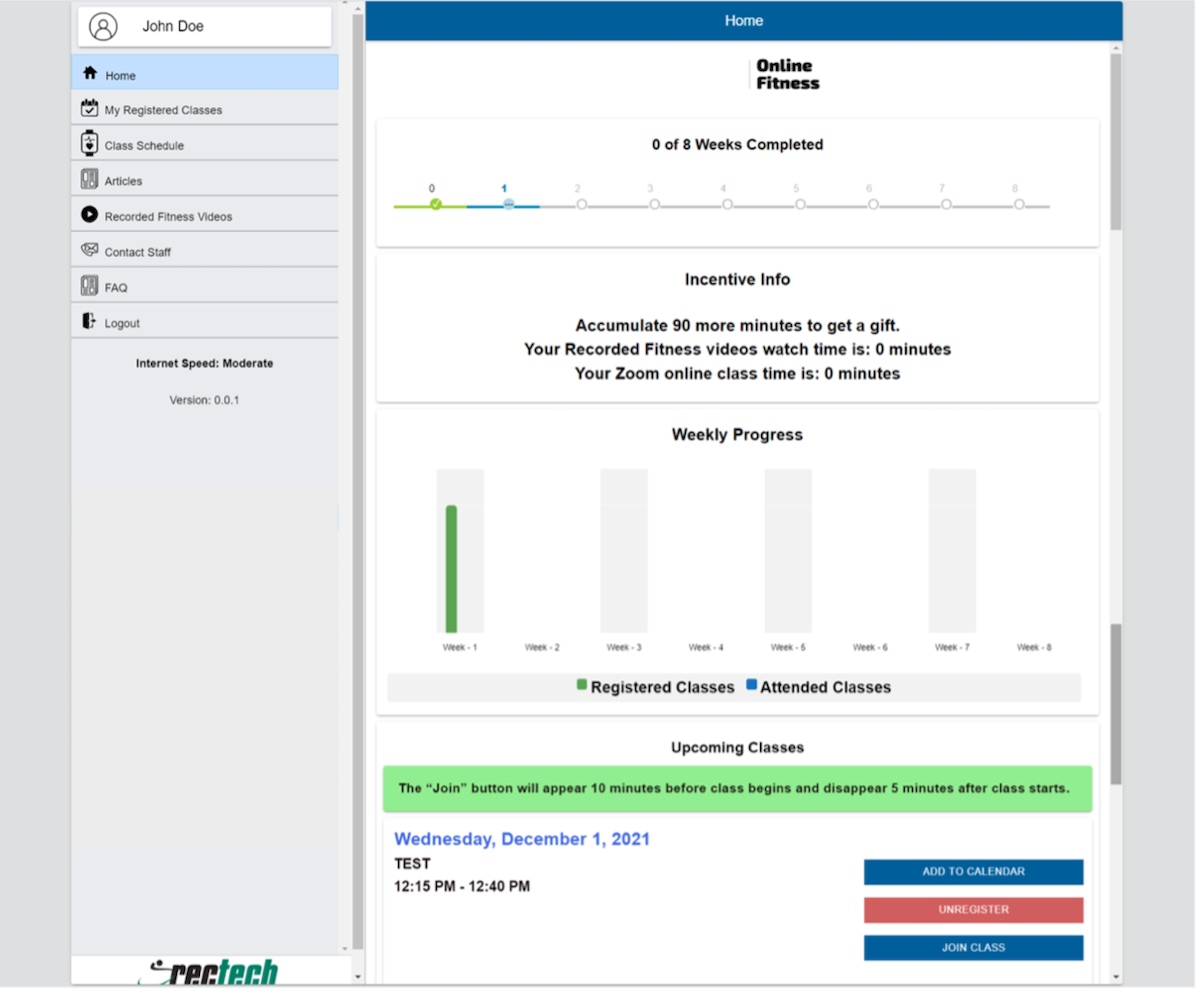
Home page of the virtual exercise platform. FAQ: frequently asked question.

The VEP is a website through which members of the fitness facility can register and attend web-based classes. This platform has features such as registering for classes up to 2 weeks in advance, a visual representation of classes registered for and attended, a library of recorded adapted exercise videos, motivational articles, contact information for staff, and frequently asked questions. The exercise platform also had a research component embedded; the members that opted to participate in the research study and qualified had the option to receive text or email reminders for classes, incentives for program adherence, access to telecoaching, and so on, as bonus features in addition to the features offered to all members. Figure 1 shows screenshots of the website home page and all existing features. The VEP was developed based on the input derived from interviews with multiple stakeholders of this project who serve in various roles at the community fitness center, including members. This needs assessment phase made it evident that the primary criteria for the success of this system would be having minimal features and a simplistic interface. This was to accommodate the community fitness facility members, who have different functional abilities and varying levels of comfort with digital technologies.

## Methods

### Design and Development

#### First Iteration: VEP Version 1

After 75% completion of the prototype development (VEP version 1), formative user testing was conducted with the community fitness center employees who are also fitness facility members, age 18 years and above, able to use a computer and communicate in English. Participants were educated about their rights and provided informed consent over the phone (Multimedia Appendix 1). During the data collection session conducted over Zoom, participants were provided access to the platform and asked to explore all features of the platform and provide feedback. Participants completed the System Usability Scale (SUS) and the Questionnaire for User Interface Satisfaction (QUIS). The SUS consisted of 10 questions with response options ranging from 1 (strongly disagree) to 5 (strongly agree). A score of 0-100 is computed using the recommended scoring instructions, with a score of 68 corresponding to a percentile ranking of 50% [14,15]. Existing literature has shown that the SUS is reliable and valid [14,16]. The QUIS was used to assess the participants’ satisfaction with the computer interface [17]. The QUIS consisted of 21 questions on a 10-point scale, with responses ranging from 0 (various adjectives describing the task as least positive) to 9 (various adjectives describing the task as most positive). Questions on the QUIS were grouped into 5 sections, including overall reactions, screen, terminology and system information, learning, and system capabilities. A mean score was computed for each section. The QUIS has been found to be reliable with satisfactory validity [17].

Seven employees of the fitness center, ages 26-62 (mean 43) years, were recruited through word of mouth. The average usability score reported on the SUS by the participants was 91.79 out of 100, with scores ranging from 75 to 100. Average participant satisfaction with the user interface measured using the QUIS was 9.41 out of 10. Specifically, the average overall reaction to the software was 8.89; screen design and layout, 9.69; terminology, 9.49; learning, 9.61; and system capabilities, 9.71. Most of the participant feedback focused mainly on language changes. For example, the majority preferred using “my registered classes,” and “recorded fitness videos” instead of “classes” and “on-demand videos.” Participants suggested that text be added to the FAQs in addition to the FAQ videos, and one participant suggested adding descriptions of the video playlists instead of clicking each video to find out what it was. Another suggestion was to change the font of the research consent page for better readability for people with visual impairments. Some of the feedback was to add more instructions to progress to the next step. For instance, on the page where participants are introduced to research, individuals cannot move to the next page without watching the research overview video. A few participants were unclear on this and suggested that language be added at the bottom of the page: “Watch our video to learn more about the Online Fitness Study.” One participant had trouble entering their date of birth on the sign-up page on their Chromebook.

#### Second Iteration: VEP Version 2

All the suggestions and issues identified were addressed in the next version of the system. The final product (VEP version 2) was launched in October 2021. Another formative evaluation of this version, VEP version 2, was completed with the community fitness facility members. The study design was informed by the Staggers Health Human Computer Interaction Framework, in which users (providers or patients) exchange information with technology by initiating specific tasks and responding to outputs from the system [18]. The exchange is influenced by the characteristics of the users and the functionalities and representations of the system [18]. To investigate these interactions, this usability evaluation study recorded all participants’ behaviors as they interacted with the VEP through realistic use-case scenarios, completing common tasks a user would wish to perform. Research suggests usability testing with 5 users is acceptable [19-21].

### Participants

Participants were recruited from the community fitness center through word of mouth, using the same sample for both strands. Eligibility criteria for VEP version 2 testing were based on the following inclusion and exclusion criteria: (1) the participant should have membership in the community fitness facility and have not used the VEP, (2) be 18 years or older, and (3) be fluent in English. Participants in this group were provided US $20 compensation for their time.

### Mixed Methods Design

**Figure 2:**
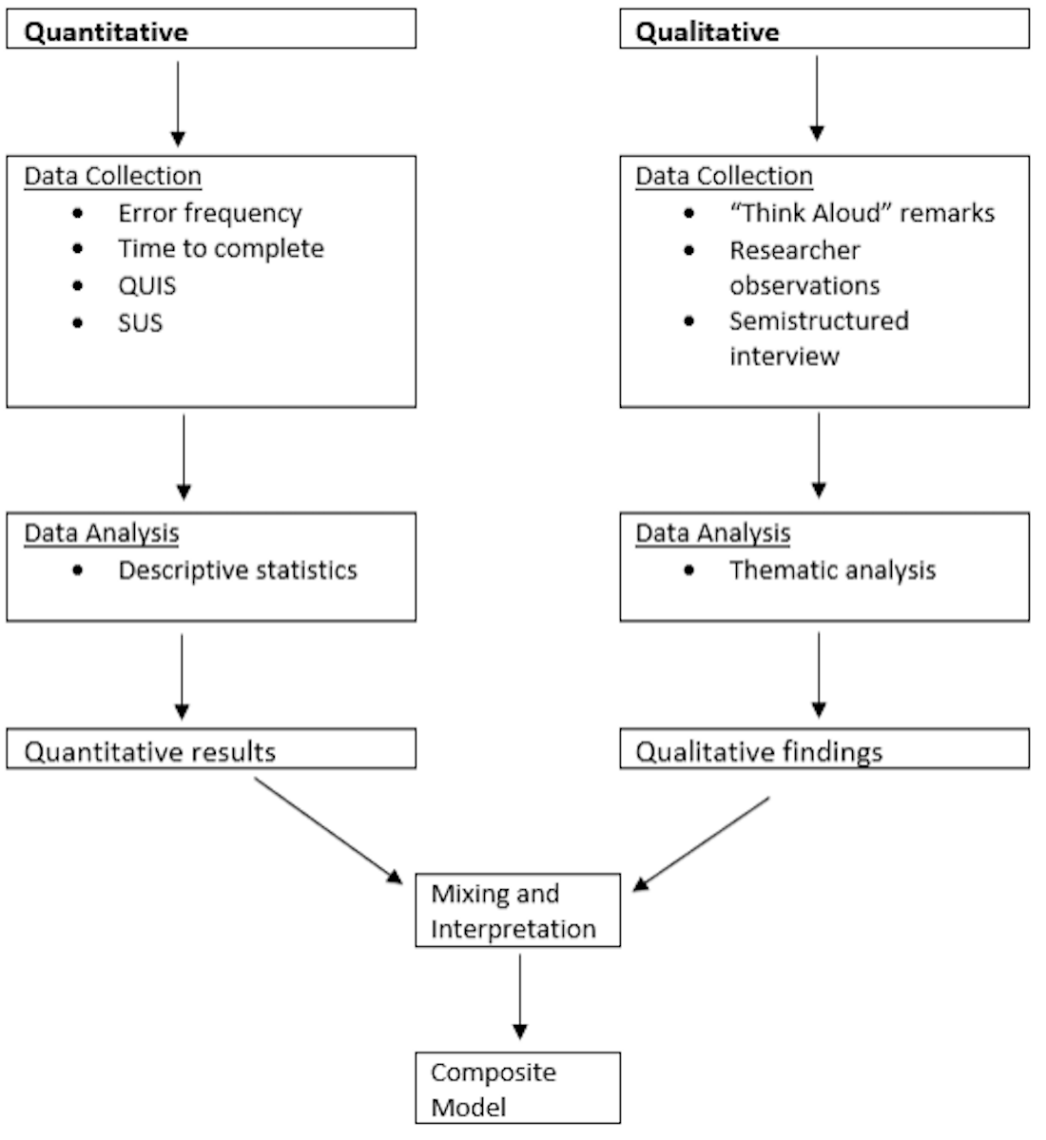
Concurrent mixed methods design procedural diagram for virtual exercise platform (VEP) version 2.0. QUIS: Questionnaire for User Interface Satisfaction; SUS: System Usability Scale.

The rationale for using mixed methods research is to use an expanding integration strategy to better understand central phenomena using 2 sources of data that expand on different aspects [12,22]. The mixed methods intent in data collection integration was to compare and expand upon both strands. An independent intramethod strategy was used for both strands, where each source of data was examined using the appropriate analysis, and then findings were integrated to form overall interpretations [23]. A diagram of our procedures can be found in Figure 2. Integration occurred during data interpretation through the use of a joint display [12].

### Data Collection

Remote, real-time, moderated user testing was conducted through Zoom (Zoom Video Communications), and participants joined from their home or work in a private location. At the start, participants were again informed that participation is completely voluntary, that they can revoke consent at any point during testing, and that no personally identifiable participant information will be disclosed or shared outside the research team. Next, the participants completed a pretest questionnaire about demographic information and their comfort with using technology in general. Finally, the purpose of the usability evaluation study and an introduction to the general testing methods and tasks were presented by the researcher. A moderator script was used to ensure consistency throughout all sessions (Multimedia Appendix 2). Even though the website is accessible via mobile devices, testing was conducted on desktop devices due to logistical reasons. The entire Zoom session was recorded for data analysis purposes. The testing was conducted on the staging server to avoid workflow disruption on the production server.

Participants in VEP version 2 were provided access to the platform and presented with 3 scenarios composed of a series of tasks a typical user would wish to perform. All scenarios were pretested by the researcher to determine optimal workflow and get an estimate of task time. The scenarios and tasks were as follows:

#### Scenario 1

Participant signs up for the VEP and agrees to participate in research:

- Task: Create a user account and login.
- Task: Consent to participate in the research component and complete the preintervention survey.
- Task: Register for the “TEST class” and “ABCs of Balance” class.
- Task: Add the class to the calendar.
- Task: Join the “TEST” class.

#### Scenario 2

Participant signs up for the VEP but does not agree to participate in research:

- Task: Create a user account and login.
- Task: Decline to participate in the research.
- Task: Register for the “TEST” and “ABCs of Balance” classes.
- Task: Add the class to the calendar.
- Task: Join the “TEST” class.

#### Scenario 3

Participant signs up for the VEP but does not agree to participate in research. Later joins the study.

- Task: Create a user account and login.
- Task: Decline to participate in the research and later join the research.
- Task: Register for the “TEST” and “ABCs of Balance” classes.
- Task: Add the class to the calendar.
- Task: Join the “TEST” class.

In addition to these tasks, participants were asked to navigate the home page features and share their thoughts and understanding.

### Measurements

A concurrent mixed methods approach was used for data collection and analysis. As the participants engaged in the described scenarios and tasks, several quantitative and qualitative observations were made. In such an approach, the effectiveness of the system is indicated by the solution quality and error rates. The efficiency of the system is indicated by the learning time and time taken to complete the task. Satisfaction can be measured by attitude rating scales [13].

In this study, we used the number of errors encountered while performing tasks, task success (whether it was done easily, with difficulty, or incompletely) to indicate effectiveness, and task completion time for efficiency. In addition, participants completed the SUS survey to evaluate usability and the QUIS survey to measure satisfaction.

The “concurrent think aloud” method was encouraged by the moderator to gauge the participants thoughts as they worked through the testing [24]. Once the scenarios were completed, SUS and QUIS surveys (Multimedia Appendix 3) were administered through Qualtrics to the participants to numerically rate their overall satisfaction with the site [16,25]. The QUIS survey covers the overall reaction to the software, screen, terminology, system information, learning, and system capabilities.

Following completion of the surveys, each participant underwent a semistructured interview (5-15 minutes) to further gauge their subjective thoughts on using the system. They were also asked for suggestions for improving of the site.

### Data Analysis

Quantitative data (effectiveness, efficiency, and satisfaction) were analyzed with descriptive statistics, and qualitative data, including recordings, moderator observations, responses from questionnaires, and interview results, were transcribed and coded. Common themes were derived from the qualitative data.

### Ethics Approval

The study was approved by the University of Alabama at Birmingham’s institutional review board (IRB-300006060).

## Results

### Formative Evaluation of VEP Version 2

Five members, ages 36-78 (mean 54) years, were recruited through word of mouth. Table 1 provides details of the participants’ demographics, which include the type of disability, highest level of education, field of work, and years of work experience. On a scale of 1-5, with 1 being the least comfortable and 5 being the most comfortable, participants reported their comfort level with using new websites. The device used by the participant during testing was also captured.

**Table 1:** Participant demographics.

ID	Age (years)	Sex	Disability	Highest level of education	Field of work, education	Years of work experience	Device used for testing	Comfort level exploring new websites (1 to 5)
P1	36	M^a^	SCI^b^ and above knee amputation	High school	Ortho and prosthetic technician	6	Desktop computer and cell to talk	3
P2	42	F^c^	Stroke (left side paralysis)	College degree	Nursing	7	Chromebook	4
P3	52	M	SCI (C5/C6 quadriplegic)	High school	Banking	20	Laptop	5
P4	62	M	Incomplete SCI (C6/C7)	BS	Engineering	30	Laptop	5
P5	78	F	Old age (pain in the foot and difficulty walking)	PhD	Psychology	26	Laptop	5

^a^M: male.

^b^SCI: spinal cord injury.

^c^F: female.

### Effectiveness

The results of task effectiveness are presented in Table 2. All 5 participants completed were able to create an account and login (tasks 1 and 2) with ease. Participant 1 did not go through the research path, so completing the preintervention surveys (task 5) was not applicable for them. Participant 2 completed the preintervention surveys with ease, while participant 3 had some difficulty selecting the sliding bars in the surveys and needed help to complete the task. All 5 of them did not complete task 4 (adding an .ics calendar file to their personal calendars).

**Table 2:** Task effectiveness of each participant.

Task	# Errors; task difficulty				
	P1	P2	P3	P4	P5
1. Create an account and login	0; Easy	0; Easy	0; Easy	0; Easy	0; Easy
2. Register for a class	0; Easy	0; Easy	0; Easy	0; Easy	0; Easy
3. Join class	0; Easy	1; Difficult	0; Easy	0; Easy	0; Easy
4. Calendar	—^a^	—	—	—	—
5. Complete surveys	N/A^b^	0; Easy	2; Difficult	0; Easy	0; Easy

^a^Incomplete.

^b^N/A: not applicable.

Completing the surveys (task 5) had the largest number of errors (2), which were committed by the same participant (P3), while task 1 and task 3 had 1 error each by one participant (P3 and P2, respectively). Specific errors included not fulfilling the password character requirement, clicking the wrong screen element to join a class, and confusion related to sliding bars in surveys.

### Efficiency

Efficiency was assessed by recording how many seconds it took participants to complete each task. Table 3 provides the time taken to complete each task by each participant. The average time to create an account and login was 82.2 seconds, with a range of 55-110 seconds. Registering for a class and joining a class took the least amount of time, at 11 and 9.2 seconds, respectively. Adding a class (.ics file) to their calendar was not completed by any of the participants. Completing the surveys took an average of 921.5 seconds.

**Table 3:** Time each participant spent completing a task.

Task	Time (seconds)					
	P1	P2	P3	P4	P5	Mean (SD)
1. Create an account and login	110	99	71	55	76	82.2 (22.12)
2. Register for a class	8	12	4	21	10	11 (6.32)
3. Join class	4	27	5	5	5	9.2 (9.9)
4. Calendar	—^a^	—	—	—	N/A^b^	N/A
5. Complete surveys	N/A	930	958	840	958	921.5 (55.9)

^a^Did not complete.

^b^N/A: not applicable.

### Satisfaction

**Figure 3:**
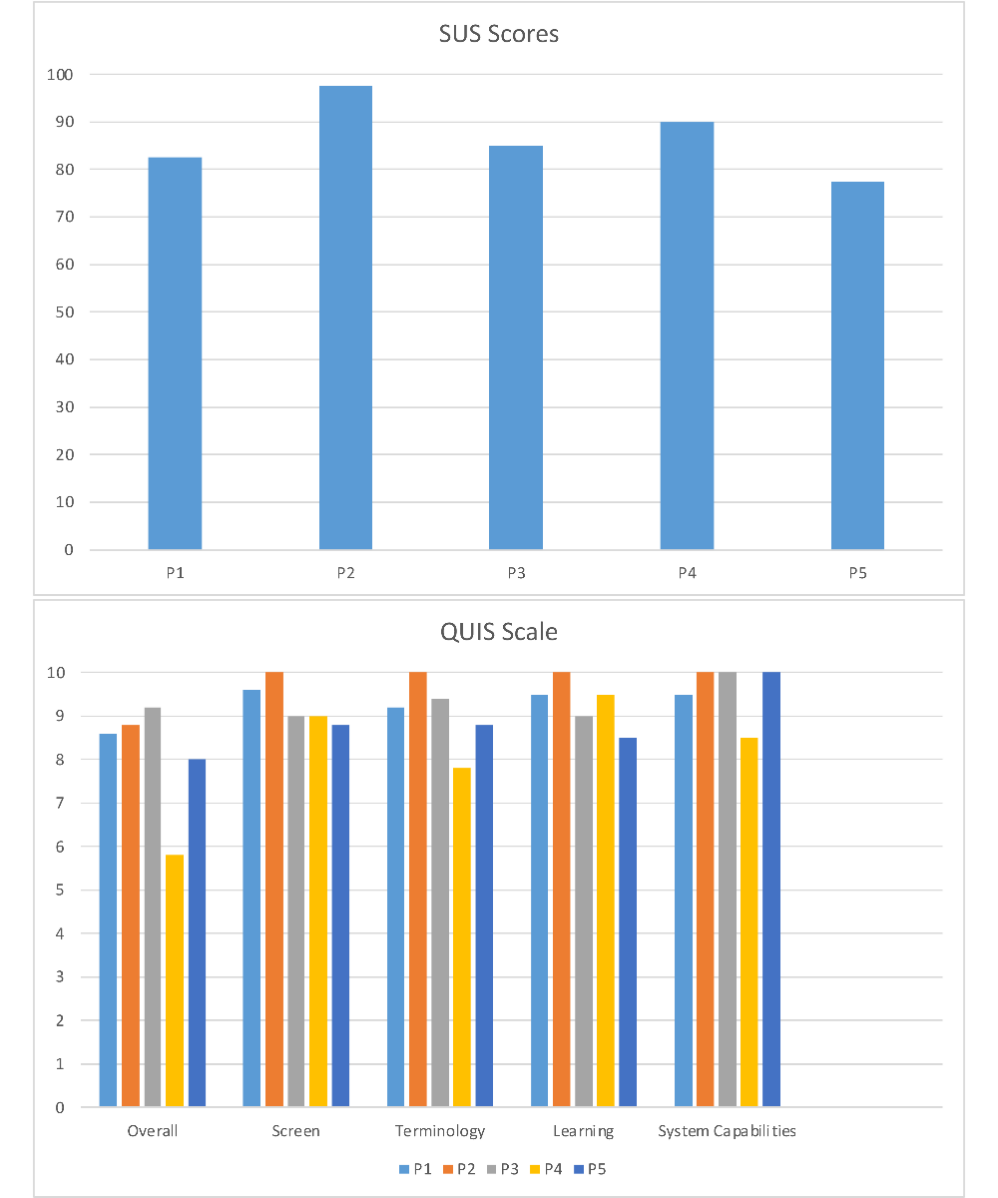
Computed SUS scores for each participant (top) and average QUIS section scale scores for each participant (bottom). QUIS: Questionnaire for User Interface Satisfaction; SUS: System Usability Scale.

Data for the SUS and QUIS are shown in Figure 3. The average satisfaction score reported using the SUS was 86.5 out of 100 across the 5 participants, ranging between 77.5 and 97.5. The average satisfaction with the user interface measured using the QUIS was 8.08 out of 10. Specifically, the average overall reaction to the software was 9.28, screen and terminology and system information were 9.04, learning was 9.3, and system capabilities were 9.6.

### Usability Findings and Recommendations

#### “Add to Calendar” Button

**Figure 4:**
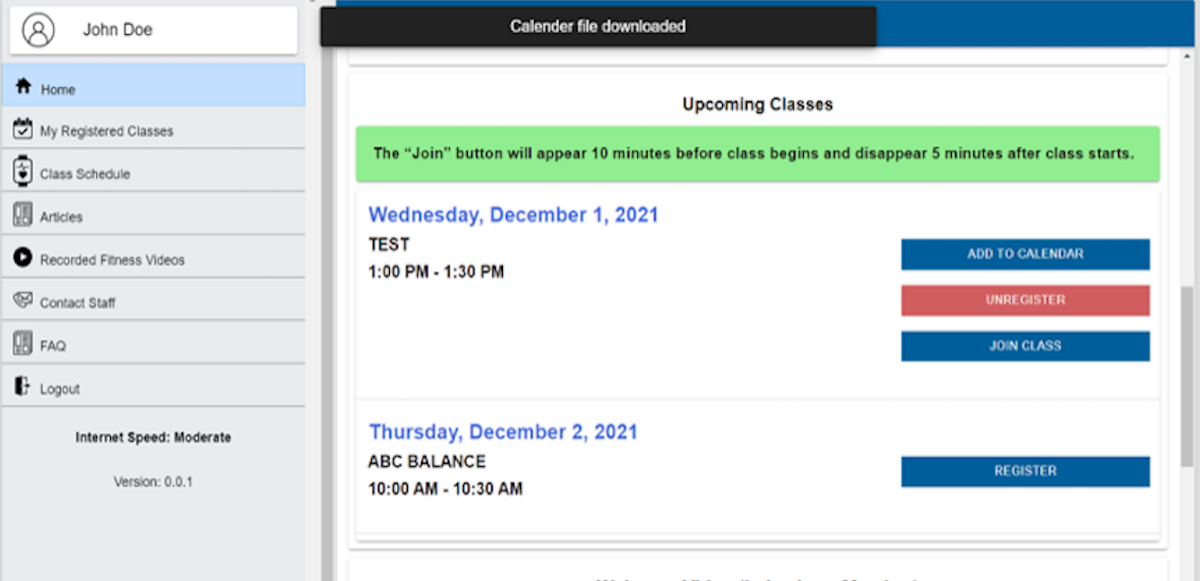
Home screen with “Add to calendar” feature and “calendar file downloaded” message on top. FAQ: frequently asked question.

As soon as the participant registers for a class, “Add to calendar” appears on the screen (Figure 4). When that button is clicked, an .ics file is downloaded to the participant’s device that must be clicked to add an entry to the participant’s personal calendar. A pop-up message “calendar file downloaded” also appears on top (Figure 4). During testing, the calendar feature had technical glitches for participant 5 and could not be tested. The remaining 4 participants were confused about the “Add to calendar” feature. When the file was downloaded during the task, participants were not sure what to do with it. One of them said,

I was expecting something different not a tab that drops down to say download. I was looking for some tab that directly adds to your calendar.

After the researcher explained what should be done, another participant thought it was a great feature. The participant commented that they were using a relative’s computer and thus did not click on the downloaded .ics file.

#### “Join Class” Button

This button appears 10 minutes prior to the class start time and disappears 5 minutes after the class starts. This was a requirement from the program staff during the needs analysis to make sure the class was not disturbed by late entrants to the classes. During the testing, 4 participants did not have any problem locating it. One participant (#2) had some confusion as they were expecting consistent coloring for the highlighted message and join button (“Can it be green as the banner says join button? I will typically look for green button that says join”).

#### Survey Slider Scales

**Figure 5:**
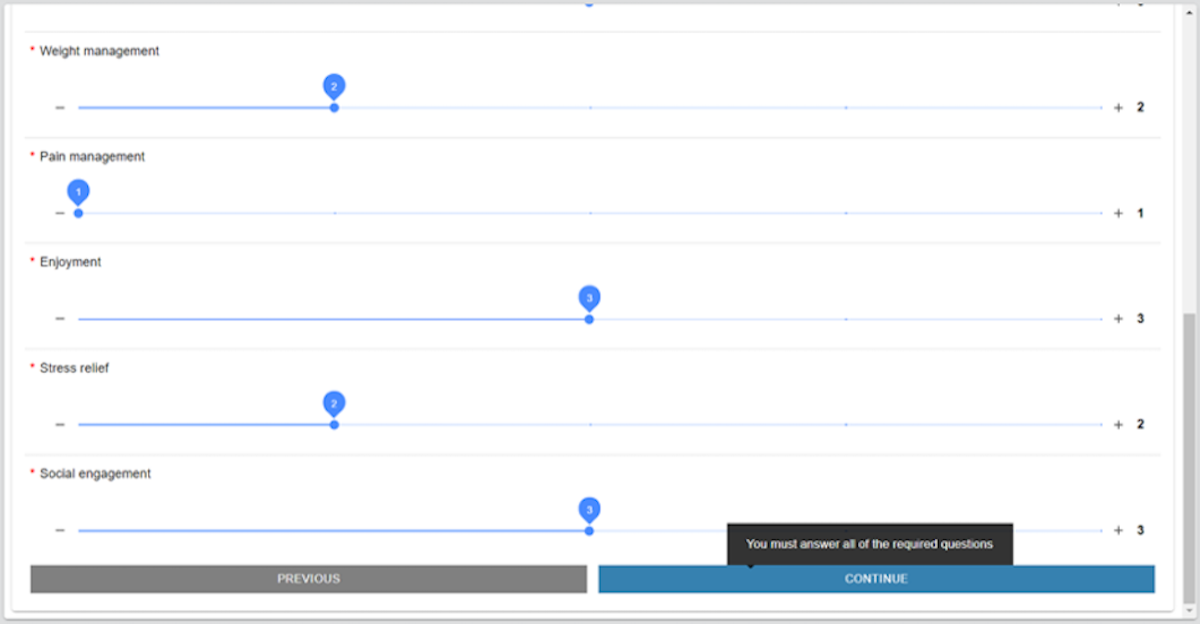
Preintervention survey slider scale.

The preintervention research questions that use a slider scale were preset for the minimum value of the scale. However, the scale had to be clicked to indicate to the system that the user responded, even if the participant’s response was that of the minimum value of the scale. Simply not touching the slider due to the preset value of the slider being equal to the value the user wanted to respond with resulted in the response not being detected (Figure 5). One of the participants (#3) had trouble moving to the next page, as some of their answers were minimum values (0 or 1), and they assumed that they had answered every question on the page. This resulted in the participant getting frustrated after a few attempts. Once hints were provided by the researcher, the participant was able to figure out the technique to respond (“If the survey would highlight that question was not answered that would have helped instead of going up and down the page on what went wrong”; “If I knew exactly which question it was, that would have helped”).

#### Button Placements

**Figure 6:**
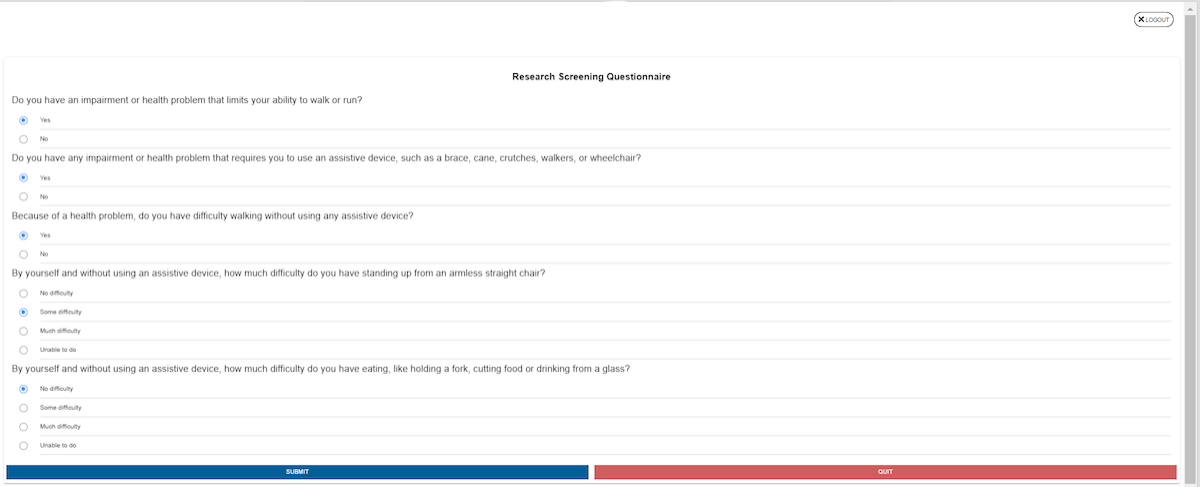
Shows placement of the “Submit” button.

One participant (P2) had confusion picking the submit button in the prescreening screen. They explained that they are used to positive buttons placed on the right side of the window (Figure 6). “Usually when you take surveys, they are (the positive buttons) swapped in other surveys that you take for something different.”

### Qualitative Results

All 5 participants agreed that they would recommend the system. Some of the themes that came out of the think-aloud protocol and the follow-up interviews indicate that, overall, the system is simple, accessible, and user-friendly. Participants also mentioned their least favorite and most favorite parts of the system and gave suggestions on what else could be improved in the system.

Representative quotes from the interviews are provided below.

1. Simple: “I think it is pretty good like it is now. If you start putting too much stuff on there, you know people will lil overwhelmed.”
2. User-friendly: “I like the system. If I want to join a class it will be easy to find one in the timeframe that would work for me and sign up for and if something happens or if I double book for, I can go back and unregister.”
3. Accessible: when asked if they thought the system was accessible for people with disabilities, all 5 said “yes.” “Ya, I have no finger function. I could navigate through it and type messages and stuff in it. So, I think. … If somebody with my level of injury can navigate through it, anybody can do it. Might be more difficult on a cell phone.”
4. Favorite and least favorite parts of the system: participant 2’s favorite part was the contact feature, as it avoids directly having to reach out to or making a phone call to staff. On the contrary, this was participant 1’s least favorite part, as they thought that many staff could read this message and it would not be private. Participant 1 liked the articles and classes, while “add to calendar” was the least favorite feature for them. Participant 3’s favorite part was the simple way to register for classes and the availability of adapted classes, and their least favorite part was the length of the preintervention surveys and some of the survey questions related to mental health aspects. They thought that the questions were intrusive. “I felt mental health questions were little invasive... I didn’t see the need of that for a workout program. Other than it was very clear and to the point, and I think it is going to be a very good program.”
5. To be improved: When the participants were asked what they thought should be improved in the system, they said they like it as it is. However, when the researcher asked for future changes to take the system to the next level, some of the suggestions were: (1) options to include one-on-one class sign-up, (2) offering classes for different levels from beginner to advanced, (3) adapted sports videos as part of recorded videos, (4) a revised add to calendar feature, (5) highlighting the question that was not answered in the survey to proceed further, and (6) more company branding throughout the website.

### Mixed Methods Integration

The joint display for this study can be found in Table 4. The effectiveness of the system could be improved by making changes to the survey sliding scale feature. However, the surveys are secondary outcomes only used for research purposes. Refining the calendar functions and revising important buttons in the system would enable the users to be more efficient with interaction. Our participants were highly satisfied with the current state of the system because they felt it was simple to use, did not overwhelm them, and presented exercises that were adequately adapted for them and their peers. The combination of quantitative data and qualitative findings suggests that our participants found the system highly usable.

**Table 4:** Joint display comparing quantitative results with qualitative findings by participant.

Participant	Effectiveness			Efficiency			Satisfaction			Usability	
	Total errors	Researcher observation	Seconds to complete		Researcher Observation	QUIS^a^ score		Illustrative quote	SUS^b^ score		Illustrative quote
P1	0	The participant was able to navigate the system easily.	Prescreening surveys was not applicable		The participant was confused about the calendar feature.	8.5		“I think this is a really great way to get people back into their normal existence in life, get them strong again and get them back out.”	82		“System is easy to navigate, pretty straightforward, everything looks well, big enough font, easy to understand and move around.”
P2	1	The participant had difficulty finding the “Join class” button and did not understand how the calendar feature works.	1068 seconds		The participant needed help to complete the prescreening questions. The participant also had confusion selecting between the “Submit” and “Quit” buttons in the survey. When asked, the participant responded that it is usually swapped in other systems.	8.8		“I can check recorded videos for fitness even if I don’t have time to register for classes.”	98		“I think it’s good. Something that tells you about what’s going on in the home page and also the visual. That’s easy to follow.” [The participant’s comment about frequently asked questions]
P3	2	The participant navigated the system easily but needed help with completing the surveys.	1038 seconds		The participant was confused about the calendar feature and suggested to have text reminders instead of email.	9		“Being a quadriplegic there are certain exercises, I won’t be able to do… sounds like they would try to adapt it different disabilities.”	84		“If somebody with my level of injury can navigate through it, anybody can do it.”
P4	0	The participant navigated the system easily.	921 seconds		The participant had a road block at one point of the survey but quickly understood the system and completed the task.	5.8		“Home page pulls everything together in one spot, ease of registration, menu layout was clear.”	90		“The system is functional and basic. Intuitively obvious to the casual observer. One thing you could do is kind of have like your account where you can edit your information.”
P5	0	The participant navigated the system independently and handled minor glitches with the surveys.	1049 seconds		Participant navigated the system easily. However, recommended us to offer training sessions for people who needed help. Have a training session so they need not figure out how to use it by themselves.	8.0		“For lot of people it might be really helpful for people who have difficulty getting there, for people in remote situations.”	78		“May be adaptations needed, like my friend with vision problem.”

^a^QUIS: Questionnaire for User Interface Satisfaction.

^b^SUS: System Usability Scale.

## Discussion

The findings from this study show that the evaluated website is usable, accessible, and acceptable to people with disabilities. This is evident from the SUS score of 88.3, which is considered excellent [26], as well as the high QUIS scores. When asked what could be improved in the system to get a higher score, one participant stated:

Honestly, I was going to put 10 but there is always room to improve. In my mind it was already at 10. I didn’t want to look like I was clicking 10 on everything. I pretty much felt like everything was 10 to be perfectly honest with you.

Based on what could be improved, many are futuristic features about the content. A few features, like the calendar and surveys, were recommended by the participants to be improved.

Even though the time to complete the task was noted, it was not compared with a baseline time. This is since the time taken to complete the task can vary across people with different functional abilities due to their disability. However, this time taken to complete a task in addition to effectiveness tasks was compared with the researcher’s observation and qualitative interviews.

**Figure 7:**
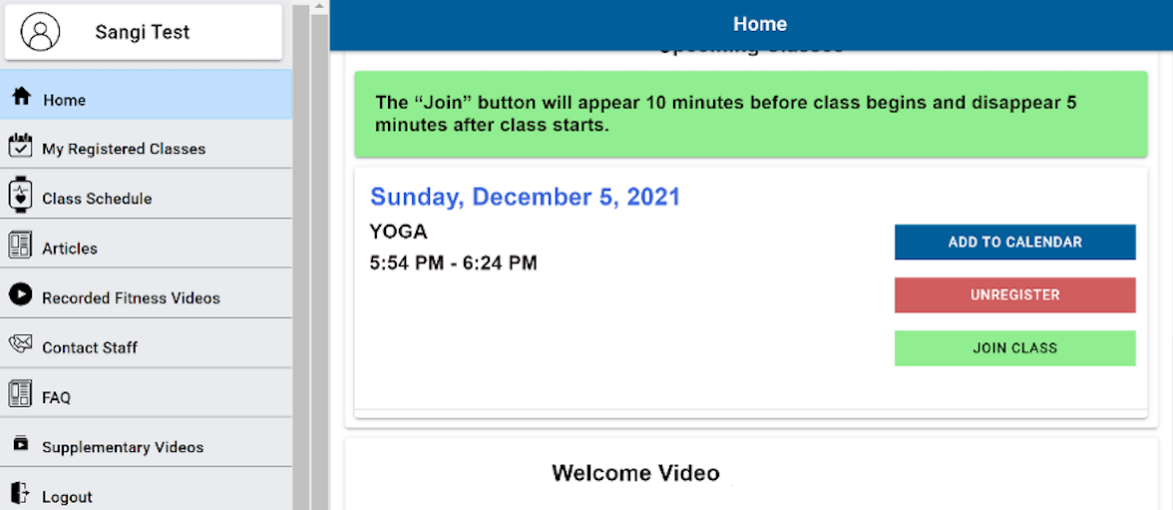
Recommended color change to the “Join class” button. FAQ: frequently asked question.

Despite the system’s effectiveness, there is still room to tweak the system further. Based on the usability findings, the following are the recommendations to improve the system:

1. Add to calendar: A detailed instruction to add the calendar can be added as a tool tip to educate the participants. For scenarios like when a user has to use someone else’s device to register, the .ics file can be emailed to the participant as soon as they register.
2. Change the color of the “join class” button to green (Figure 7). This will be consistent with the message about the join button and will avoid confusion for the participants.
3. On the survey slider scale, an error prevention strategy can be used to avoid going up and down to identify which question was not answered.

**Figure 8:**
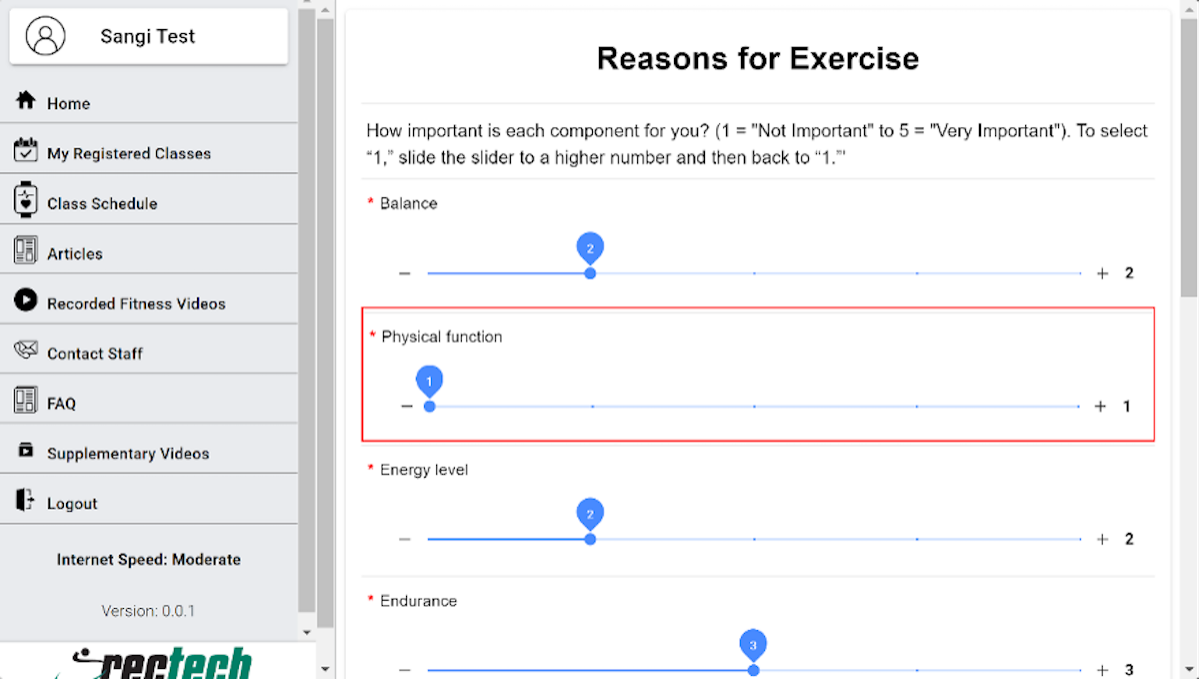
Recommended changes to highlight unanswered questions in the slider survey. FAQ: frequently asked question.

Highlighting the question that is skipped or not answered using a blinking box is a potential strategy (Figure 8). In addition, instructions on the top of the page about answering all slider questions could be used.

### Strengths and Limitations

This usability evaluation study was conducted with actual end users and was based on the ISO usability standards by means of validated instruments and a mixed methods approach. Limitations included not formally conducting usability testing on mobile devices with end users and a lack of a larger number and wider range of participants (older adults, people with visual impairments, and people less comfortable with technology). Participants completed the satisfaction surveys during the Zoom call, which could have resulted in bias.

### Conclusions

The virtual exercise fitness platform meets the needs of people with disabilities with high SUS and QUIS scores and is efficient and effective. Future studies should test with a larger number and a wider range of participants across different devices.

## Appendix

Multimedia Appendix 1 Information sheet (Consent).

Multimedia Appendix 2 Moderator script for the interviews.

Multimedia Appendix 3 Usability surveys and general qualitative questions that were completed by the participants.

## Abbreviations

ISO: International Organization for Standardization
QUIS: Questionnaire for User Interface Satisfaction
SUS: System Usability Scale
VEP: virtual exercise platform
