# Supplementary material for: Development and Formative Evaluation of a Virtual Exercise Platform for a Community Fitness Center Serving Individuals With Physical Disabilities: Mixed Methods Study
Source: JMIR Form Res. 2023 Dec 15;7:e49685. doi: 10.2196/49685 (PMC10757225; doi:10.2196/49685)
Supplement: Multimedia Appendix 1 [file formative_v7i1e49685_app1.docx]

**Title of Research:** *RERC on Exercise and Recreational Technologies for People with Disabilities (RECTECH IV) (Usability of Virtual Exercise Support System for people with disabilities)*

**UAB IRB Protocol #:** IRB-300007079

**Principal Investigator:** Mohanraj Thirumalai, Ph.D.

**Sponsor:** NIDILRR (National Institute on Disability, Independent Living, and Rehabilitation Research)

We are asking you to take part in a research study. The purpose of this protocol is to evaluate the usability of xxx’s virtual exercise support system to facilitate exercise sessions for people with disabilities. This study will enroll up to 20 people who are xxx members.

You will be scheduled to join a zoom call with the study PI and research staff at a time that is convenient for you. During the call, PI will collect your demographic details and you will be assigned a study id. You will be referred by that id in the user testing process.

First, the PI will ask you to share your computer screen and give you tasks to perform on the virtual exercise support system platform. Sample tasks will include login to the system, navigate to different pages, register for classes. You will be asked to speak your thoughts while performing the tasks (i.e., thinking aloud). The testing procedure will be recorded. A research assistant will take written notes from their observations of you during the usability tasks, including whether you completed the tasks and how long it took.

Second, you will be asked to fill out two short surveys that will help us to determine the usability of the system. Third, the PI will interview you to learn about your perceptions about using technology to receive exercise programs, likes/dislikes, challenges, and usefulness of contents of the system. Entire testing and interview will be recorded and transcribed for research purposes. During the recording, you will be referred by your study id number and your information will be deidentified.

You may not benefit directly from taking part in this study. However, this study may help us better understand your experiences and perceptions that will inform us to improve the design and features of the system.

Information obtained about you for this study will be kept confidential to the extent allowed by law. However, research information that identifies you may be shared with people or organizations for quality assurance or data analysis, or with those responsible for ensuring compliance with laws and regulations related to research. This includes (1) the UAB Institutional Review Board (IRB). An IRB is a group that reviews the study to protect the rights and welfare of research participants; (2) NIDILRR; (3) the Office for Human Research Protections (OHRP).

All information (e.g., survey response, written records of interviews), and audio transcripts will be kept on a password protected computer on a central server at UAB. The audio recordings will be destroyed after data is analyzed and published. Everything you talk about will be confidential and your name will be removed from any written script. There is a slight risk of loss of confidentiality, but we will record information so that you will not be easily identified. The information from the research may be published for scientific purposes; however, your identity will not be given out.

Participation is voluntary. There will be no penalty if you decide not to be in the study. You are free to withdraw from this research study at any time. Your choice to leave the study will not affect your relationship with this institution. You are also free not to answer any specific question you do not wish to. If at any time you wish to terminate the interview, simply state that you want the interview to stop. The researcher will stop the interview immediately. If you wish to withdraw completely from the study, tell the researcher in person. The audio recording and the written records of your interview will be immediately destroyed.

The alternative is not to participate.

If you are an employee of xxx, taking part in this research is not a part of your works or duties. You can refuse to enroll, or withdraw after enrolling at any time before the study is over, with no effect on your job at UAB or xxx. You will not be offered or receive any special consideration if you take part in this research.

There will be no cost to you for taking part in this study. You will be given a $20 gift card as a compensation for your time.

If you have any questions, concerns, or complaints about the research please contact Dr. Mohanraj Thirumalai at XXX-XXX-XXXX or after hours by emailing him at mohanraj@uab.edu.

If you have questions about your rights as a research participant, or concerns or complaints about the research, you may contact the UAB Office of the IRB (OIRB) at (205) 934-3789 or toll free at 1-855-860-3789. Regular hours for the OIRB are 8:00 a.m. to 5:00 p.m. CT, Monday through Friday.
