# Supplementary material for: Development and Formative Evaluation of a Virtual Exercise Platform for a Community Fitness Center Serving Individuals With Physical Disabilities: Mixed Methods Study
Source: JMIR Form Res. 2023 Dec 15;7:e49685. doi: 10.2196/49685 (PMC10757225; doi:10.2196/49685)
Supplement: Multimedia Appendix 2 [file formative_v7i1e49685_app2.docx]

Thanks for participating in the study. We went over the details with you during consenting. I would like to reiterate that these testing are for the system and not for you. There are no right or wrong answers. You are helping us to improve the system by giving your honest feedback about the system. I will be collecting some demographic information first and then give you few tasks to complete in our website. I will ask you to share your screen so some observations can be made. You are requested to think aloud while you perform the tasks. If anything is confusing or difficult, please let me know.

Once you are done with the tasks, I will give you two surveys to complete and ask few questions to get your feedback about the system.

Do you have any questions before we start?

Participant Demographics

- Name:
- Age:
- Gender:
- Disability:
- Highest level of education:
- Field of education:
- Years of work experience:
- How would you rate your comfort level using a new website on a scale of 1 to 5 with 1 being the lowest and 5 being the highest?
