# Supplementary material for: Development and Formative Evaluation of a Virtual Exercise Platform for a Community Fitness Center Serving Individuals With Physical Disabilities: Mixed Methods Study
Source: JMIR Form Res. 2023 Dec 15;7:e49685. doi: 10.2196/49685 (PMC10757225; doi:10.2196/49685)
Supplement: Multimedia Appendix 3 [file formative_v7i1e49685_app3.docx]

**System Usability Scale (SUS)**

Participants were asked to score the following 10 questions with one of five responses that range from Strongly Disagree to Strongly Agree.

Please answer the following 10 questions using the following responses:

Strongly Disagree | Disagree | Neutral | Agree | Strongly Agree

1. I think I would use the system frequently.
2. I found the system unnecessarily complex.
3. I thought the system was easy to use.
4. I think that I would need the support of a technical person to be able to use the system.
5. I found the various functions in the system were well integrated
6. I thought there was too much inconsistency in the system.
7. I would imagine that most people would learn to use the system very quickly.
8. I found the system very cumbersome to use.
9. I felt very confident using the system.
10. I needed to learn a lot of things before I could get going with the system.

**Questionnaire for User Interface Satisfaction (QUIS)**

Please rate your satisfaction of the platform you used on an ascending scale of 1 to 10.

Part A: Overall Reactions to Software

1. Systems are aesthetically pleasing
   Terrible – 1 2 3 4 5 6 7 8 9 10 – Wonderful – N/A
2. Screen designs and layout are attractive
   Difficult – 1 2 3 4 5 6 7 8 9 10 – Easy – N/A
3. Use of color combinations
   Unattractive – 1 2 3 4 5 6 7 8 9 10 – Attractive – N/A
4. System can do a great deal
   Not at all – 1 2 3 4 5 6 7 8 9 10 – Very Much So – N/A
5. System maintains one’s interest
   Never – 1 2 3 4 5 6 7 8 9 10 – Always – N/A

Part B: Screen

1. Reading characters on the screen
   Hard – 1 2 3 4 5 6 7 8 9 10 – Easy – N/A
2. Organization of information
   Confusing – 1 2 3 4 5 6 7 8 9 10 – Very Clear – N/A
3. Sequence of Screens
   Confusing – 1 2 3 4 5 6 7 8 9 10 – Very Clear – N/A
4. Knowing where you are in the task
   Confusing – 1 2 3 4 5 6 7 8 9 10 – Very Clear – N/A
5. Screen items are easy to select
   Difficult – 1 2 3 4 5 6 7 8 9 10 – Easy – N/A

Part C: Terminology and System Information

1. Terms on screen are understandable
   Confusing – 1 2 3 4 5 6 7 8 9 10 – Very Clear – N/A
2. Terms on the screen
   Ambiguous – 1 2 3 4 5 6 7 8 9 10 – Precise – N/A
3. Position of messages on screen
   Confusing – 1 2 3 4 5 6 7 8 9 10 – Very Clear – N/A
4. Instructions for commands or choices
   Confusing – 1 2 3 4 5 6 7 8 9 10 – Very Clear – N/A
5. Error Messages
   Unhelpful – 1 2 3 4 5 6 7 8 9 10 – Helpful – N/A

Part D: Learning

1. Learning to operate the system
   Confusing – 1 2 3 4 5 6 7 8 9 10 – Very Clear – N/A
2. Remembering names and use of commands
   Confusing – 1 2 3 4 5 6 7 8 9 10 – Very Clear – N/A
3. Performing tasks is straightforward
   Confusing – 1 2 3 4 5 6 7 8 9 10 – Very Clear – N/A
4. Help messages on the screen
   Confusing – 1 2 3 4 5 6 7 8 9 10 – Very Clear – N/A

Part E: System Capabilities

1. System Speed
   Too Slow – 1 2 3 4 5 6 7 8 9 10 – Fast Enough – N/A
2. System Reliability
   Unreliable – 1 2 3 4 5 6 7 8 9 10 – Reliable – N/A

**General Qualitative Questions**

These questions were open-ended and aimed to allow users to give their opinion about the system without associating it to a number. It also allowed the chance to question participants about their survey responses in order to better determine usability.

The qualitative questions may have included, but were not limited to:

- How did you like the system overall?
- Were the tasks easy to follow along?
- Why did you do [insert task] this way?
- Did you have an issue with [insert task or screen layout]?
- I noticed you paused longer than usual at [insert task], why is that?
- Did you have any other problems?
- What was your favorite part of the system?
- Would you recommend the system to anyone?
- What was your least favorite part of the system?
- Do you think you would continue to use the system?
- Why do you think the system would be usable for users with disabilities?
- Could you tell me more about [insert task or screen layout]?
- Where can the system improve?
- Do you have any questions?

**These questions were asked in no particular order.*
